# The effect of sleep disturbances on the incidence of dementia for varying lag times

**DOI:** 10.1016/j.tjpad.2024.100024

**Published:** 2025-01-01

**Authors:** Peter Alders, Almar Kok, Elisabeth M. van Zutphen, Jurgen A.H.R. Claassen, Dorly J.H. Deeg

**Affiliations:** a Erasmus School of Health Policy and Management, Erasmus University Rotterdam, PO Box 1738, Rotterdam 3000 DR, The Netherlands; b Department of Psychiatry, Amsterdam UMC, VU University Medical Center, Amsterdam Public Health Research Institute, De Boelelaan 1118, Amsterdam 1081 HZ, The Netherlands; c Department of Epidemiology and Data Science, Amsterdam UMC, VU University Medical Center, Amsterdam Public Health Research Institute, De Boelelaan 1118, Amsterdam 1081 HZ, The Netherlands; d GGZ inGeest Specialized Mental Health Care, PO Box 74077, Amsterdam 1070 BB, The Netherlands; e Radboudumc Alzheimer Center, Department of Geriatrics, Radboud University Medical Center, Nijmegen, The Netherlands; f Department of Cardiovascular Sciences, University of Leicester, University Road, Leicester LE1 7RH, United Kingdom

**Keywords:** Sleep quality, Longitudinal study, Prodromal, Epidemiology, Incident dementia

## Abstract

**Background:**

Few studies have addressed the association of sleep disturbances with incident dementia with long lag times. We add to this literature by investigating how lag times varying from 2.2 to 23.8 years affect the relationship between sleep disturbance and incident dementia in a Dutch cohort study on aging.

**Methods:**

Using eight waves of data from the Longitudinal Aging Study Amsterdam, we investigated the association of hours of sleep, difficulty falling asleep, interrupted sleep, and waking up early with incident dementia. For dementia an algorithm was used based on repeated measurements of cognitive tests and other data sources that provide strong indications of dementia. Sleep disturbances were assessed with a self-report questionnaire.

**Results:**

Of 2,218 participants, 237 (11%) developed dementia in the period 1992/3 to 2015/6. Participants ≥70 years more often reported sleep disturbances compared to those <70. Only for a short lag time (3 years), sleeping *≥*9 h was associated with incident dementia. Sleeping ≤6 h, interrupted sleep and waking up early were associated with incident dementia, particularly for lag times *≥*15 years.

**Discussion:**

We found that the association of sleep disturbances with incident dementia becomes stronger with longer lag times (particularly *≥*15 years). Studies with lag times <15 years may suffer from reverse causation due to the changes in sleep patterns caused by the prodromal phase of neurodegenerative disease. The association of sleeping ≥9 h and the incidence of dementia in analyses with a short lag time seem to be the result of reverse causation.

## Highlights

• Sleep disturbances are associated with incident dementia.
• Length of follow-up and age of sample at baseline affect the results.
• More sleep disturbance types are associated with dementia for lag times ≥*15* years.
• Association of ≥*9 h* sleep and dementia is likely result of reverse causation.

## 1 Introduction

The ageing-related increase in the incidence rate of dementia coincides with a deterioration in the quality of sleep over the lifespan. Compared to young adults, the quality of sleep in older adults is often impaired, with shorter overall sleep duration, interrupted sleep, reduced amount of deep sleep, shorter and fewer sleep cycles, and increased time spent awake throughout the night [1].

Sleep disturbances are associated with dementia [[2], [3], [4], [5]]. However, whether this association is causal, and if so, what the direction of this causality is, remains debated. Based on current evidence, the relationship between sleep and dementia is bidirectional. Poor sleep is associated with increased risk of developing dementia; vice versa, early neurodegenerative changes, preceding the development of dementia, are associated with poor sleep [6,7].

Years prior to the clinical onset of dementia, pathological changes occur in the brain. For a 70-year-old, the preclinical and prodromal phase of Alzheimer's Disease (AD) was estimated to be 17 years [8]. Therefore, the results of studies with a short follow-up that link poor sleep to an increased risk of dementia are likely to be affected by reverse causation. The few studies with follow-up periods of more than 15 years have focused mostly on the association of hours of sleep with dementia. The results from these studies are contradictory, with some studies finding an association of sleeping ≤6 h [9,10] and others *≥*9 h [11,12] with an increased incidence of dementia. Where a short period of sleep can be related to less clearance of interstitial wastes such as amyloid beta during slow wave sleep [13], the mechanisms associated with a long period of sleep are less clear. More studies with long follow-up, measuring more aspects of sleep disturbances, are needed to increase insight into the prospective relationship between sleep and dementia.

In this study we add to this strand of literature by investigating the effect of several sleep disturbances, i.e., a sleep duration of ≤6 or ≥9 h of sleep, difficulty falling asleep, interrupted sleep, and waking up early, on dementia risk over a 23-year study period, correcting for a broad range of covariates. Furthermore, because sleep behaviour might already change in the preclinical and prodromal phase of dementia, we study whether varying the time to incidence affects the relation of sleep disturbances with incident dementia. We thereby consider the age of the sample as well. Because people at higher ages have a higher risk of dementia, they have a higher probability of prodromal dementia and thus reverse causation than younger people. We hypothesized that sleep disturbances are associated with the incidence of dementia, and that some of these associations may be explained by reverse causation, specifically in older people. For example, regarding sleep duration we expected to find associations between sleep disturbances and incidence of dementia to be stronger for a sleep duration of ≤6 h of sleep when the lag time is longer and for ≥9 h of sleep when the lag time is shorter. We further expect less reverse causation in a younger population than in an older population.

## 2 Methods

### 2.1 Sample

We used data from the Longitudinal Aging Study Amsterdam (LASA), an ongoing study on predictors and consequences of changes in physical, cognitive, emotional, and social functioning of older people. The original LASA cohort is based on a nationally representative sample of adults aged 55–85 years in 1992–1993 (years of birth: 1908 – 1937, *N* = 3107), recruited in three geographic regions in the Netherlands. These regions were selected to achieve a representation of the older Dutch population. Follow-up measurement cycles were carried out every 3–4 years.

Trained interviewers visited participants in their homes. In addition to face-to-face interviews, participants who were not able or refused to participate in the complete face-to-face interview were asked to participate in a 15-min telephone interview. For participants who were not able or willing to participate in a telephone interview, a proxy respondent was asked – in a telephone interview – to answer a set of questions. The sampling and data collection procedures have been described in more detail elsewhere [14]. The LASA study was approved by the Institutional Review Board of the VU University Medical Center. All participants signed informed consent on entering the study.

Of the 3107 participants in 1992/3 (baseline), 2218 had data on sleep patterns at baseline and dementia at least one follow-up cycle. They had a first interview in 1992/3 and up to 7 interviews in follow-up cycles, with the 7th follow-up cycle in 2015/6 (maximum follow-up duration 23.8 years). Using these multiple time points for assessment of incident dementia allowed us to investigate how different lag times, the period from the measurement of sleep disturbances till the first occurrence of dementia, affect the relationship between sleep disturbance and incident dementia.

**Fig. 1:**
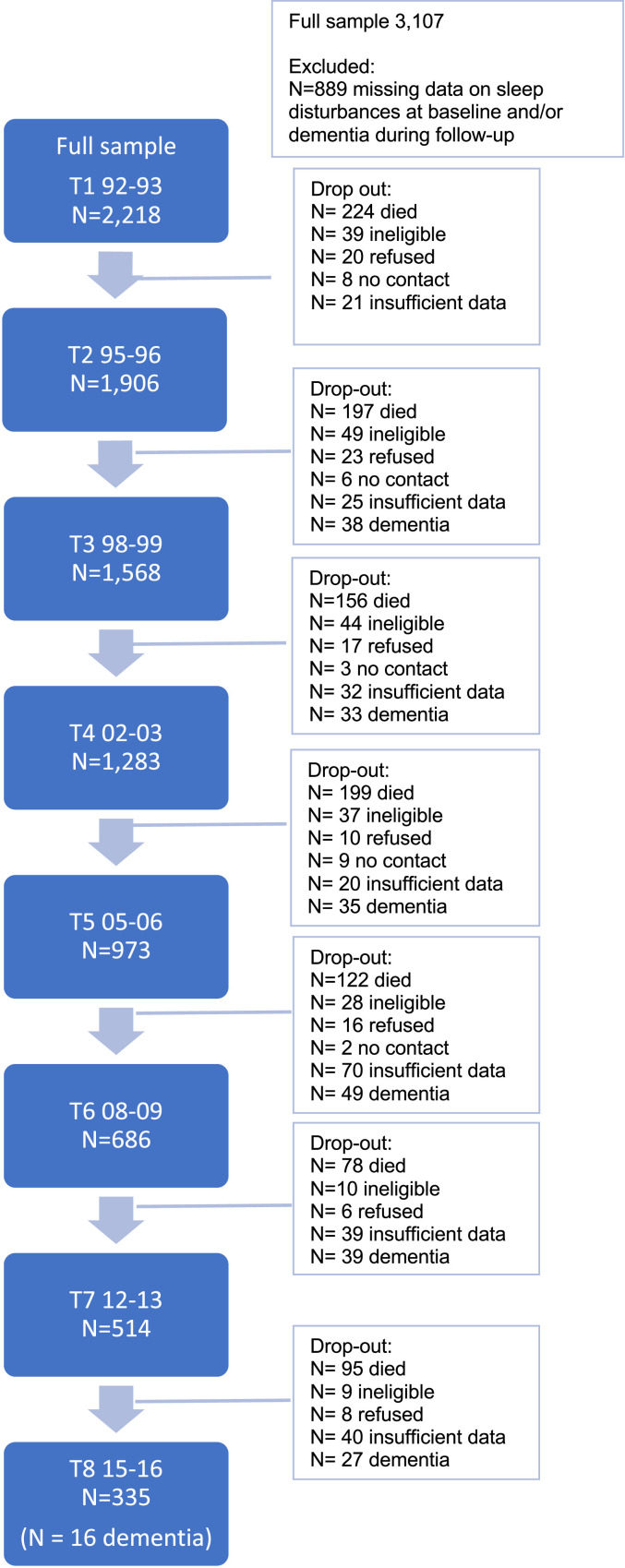
Flow chart of study sample.

Only participants without dementia at baseline were considered; observations of participants for whom the status of dementia could not be determined because of insufficient data were left out. Attrition was primarily caused by mortality (76% over a 20-year period); a relatively small proportion dropped out for other causes: frailty (15%), refusal (7%) or because no contact could be made (2%). See Fig. 1 for the Flow chart of the study sample.

### 2.2 Dependent variable: probable dementia algorithm

We applied a previously developed algorithm designed to indicate probable dementia (yes/no) at each follow-up measurement cycle [15]. The algorithm is based on five data sources.

The primary and most complete data source for determining probable dementia consists of the available measures of cognitive decline. These were either the Mini-Mental State Examination (MMSE) [16], which was administered in the face-to-face interview; a shortened telephone version of the MMSE for participants who did not participate in the face-to-face interview [15], or a shortened, 6-item version of the Informant Questionnaire on Cognitive Decline in the Elderly (IQCODE) [17] for participants for whom only a proxy interview was available. The IQCODE asks the proxy to what extent in the past 10 years he/she noticed changes in cognitive functioning in the participant in several domains. The 6 items are scored on a 5-point scale ranging from 1=much better to 5=much worse and summed up. In addition to these measures of cognitive decline, we also used comments from interviewers, general practitioner (GP) data, information on institutionalization and cause of death (obtained through Statistics Netherlands).

These data sources were combined as follows. Participants were categorized as having probable dementia if 1) the decline in MMSE score since the previous measurement wave was ≥2 SD *and* the MMSE score decreased further by ≥1 SD on the next measurement wave; or if 2) the IQCODE score was ≥28 [15]; or if 3) the interviewer recorded ‘dementia’ in their notes during the face-to-face interview; or if 4) dementia was diagnosed by a GP or a specialist; or if 5) data on institutionalization indicated whether the participant was living in a psychogeriatric nursing home; or if 6) cause of death indicated dementia as the cause of death. The status of dementia was undetermined if information was contradictory; e.g. the Mini-mental state examination (MMSE) was < 19, but cognitive decline was not persistent. Although this algorithm does not represent a clinical diagnosis of dementia, for reasons of brevity we refer to it as dementia. We set the time of the incidence of dementia halfway between the interview in which dementia was determined, and the preceding interview.

### 2.3 Independent variables: sleep

Sleep disturbances were assessed using a self-reported questionnaire. Sleep duration, based on the total number of hours of sleep as reported by the participants, was categorized in ≤6 hr/day of sleep (short sleep duration), 7–8 hr (normal), and ≥9 hr (long) because previous studies showed that both shorter and longer sleep duration are associated with onset of dementia [18]. Difficulty falling asleep, interrupted sleep, or waking up early in the morning were scored on a 4-point Likert scale. Because the wording of the first two categories (almost never or some of the time) and of the last two categories (often or most of the time) is relatively similar, we dichotomized these indicators of poor sleep quality into absent or present.

### 2.4 Covariates

Potential confounders were chosen on the basis of previous literature on the association of sleep disturbances with dementia [[9], [10], [11]]. Education was categorized into three levels: low (elementary school not completed, elementary school, lower vocational education), intermediate (general intermediate, intermediate vocational, general secondary education) and high level of education (higher vocational, college or university education). Depressive symptom severity was ascertained using the Dutch translated 20-item Center for Epidemiologic Studies Depression scale (CES-D) [19,20]. Participants were asked to indicate how often during the preceding week they had experienced each symptom, on a four-point Likert scale. The score range is 0–60, with higher scores indicating a more severe depressive symptomatology. Current physical activity was assessed using the LASA physical activity questionnaire (LAPAQ) [21]. Participants were asked how often and for how long in the previous 2 weeks they had participated in the following activities: outdoor walking, bicycling, light household, heavy household, and two additional sport activities. Based on the intensity of the activity, a total physical activity score in metabolic equivalent of task (MET) was computed (One MET unit = resting energy expenditure = 1 kcal per kg body weight per hour). Participants were asked to report their current alcohol consumption and their past and current smoking habits. Alcohol consumption was categorized into abstainers, light/moderate drinkers (1– 14 drinks/week for men and 1– 7 drinks/week for women), and heavy drinkers (more than 15 drinks/week for men and more than 8 drinks/week for women) [22]. Smoking was categorized as 0) does not smoke or stopped smoking more than 15 years ago, and 1) does not smoke but stopped smoking less than 15 years ago or 2) current smoker [23]. BMI was calculated by dividing measured body weight (in kg) by measured height (in m) squared. During the interview, participants were asked whether they had diabetes or hypertension. For hypertension, we used data of the 1995/6 interview cycle, because the question on hypertension was not asked in the baseline interview. We used self-reported data on cardiovascular disease (CVD).

### 2.5 Statistical analyses

Statistical analyses were performed with Stata 18.

To obtain insight into how sleep disturbances change with aging, we present sample characteristics stratified for age (<70 years vs. ≥70 years). In addition, we describe characteristics of participants who developed dementia, stratified for the time to dementia (within 15 years vs. after 15 years of follow-up). The cut-off of 15 years was based on the length of the typical prodromal phase found by Vermunt et al. [8].

To address the main research question, several considerations are pertinent. Because the incidence of dementia is determined at each of the 7 follow-up measurement cycles, a logical choice might be to perform the analysis with a discrete time hazard model. However, for the discrete time hazard model, we have limited power with 237 persons in whom dementia is determined across 7 follow-up periods. The alternative would be Cox regression, yet if there is an effect of dementia on sleep disturbances in the first measurement cycles, it is unlikely that the condition of proportional hazards of the Cox model would hold. Because the hazard rate of the Cox model needs to be interpreted as the weighted average of the true hazard ratios over the entire follow-up period [24], a Cox analysis of all periods provides only a global insight in the hazard ratios. Therefore, we analyzed the data in two ways. First, we investigated the effect of varying lag times by performing 7 logistic regressions with different lag times between sleep disturbances and the determination of dementia ranging from 1 to 7 interview cycles (i.e., 2.2 to 23.8 years). In Table 1, we show for each lag time how many participants from each interview cycle are included. Participants can be part of several cycles. Therefore, the number of observations varies from 7753 with a lag time of 3 years to 333 with a lag time of 23 years. Second, as a sensitivity analysis, we used the longitudinal character of the data and the multiple measurements of each respondent by performing standard Cox model regressions with lag times varying from 2.2 to 23.8 years.

**Table 1:** Number of observations in the logistic regressions with a lag time varying from 3 to 23 years.

Lag time (Years)	T1*	T2*	T3*	T4*	T5*	T6*	T7*	N
3	1906	1699	1428	1032	759	561	368	7753
6		1560	1420	1102	761	578	382	5803
9			1277	1094	784	574	383	4112
13				971	775	589	378	2713
16					682	585	379	1646
19						510	377	887
23							333	333

‘* Start date: T1 = ’92 – ’93; T2 = ’95 – ’96; T3 =’98 – ’99; T4 = ’02 – ’03; T5 =’05 – ’06; T6 = ’08 – ’09; T7 = ’12 – ’13; T8 = ’15 – ’16.

In the first set of analyses, the interview cycle when incident dementia was determined differed, but the number of interview cycles between the determination of the sleep disturbances and incident dementia was kept the same for each separate regression analysis. Thus, for each lag time of 1 to 7 interview cycles, we pooled the data. Because the data on the errors of the participants may not be mutually independent, standard errors were clustered at the respondent level.

We investigated two models: in model 1 we corrected for age at the time of the measurement of the sleep disturbances, education, and sex. In model 2, we additionally corrected for partner status, physical activity, depression, smoking, CVD, diabetes, alcohol consumption, BMI and hypertension.

### 2.6 Sensitivity analyses

We investigated the sensitivity of the results by performing a Cox regression analysis, with time since baseline as the time-scale. We varied the lag time between baseline sleep disturbances and incident dementia by ignoring measurement cycles directly following baseline. Proportional hazard assumptions were tested using Schoenfeldt residuals. In accordance with reverse causality, we found violations of the proportionality assumption.

To investigate the effect of age on the association between sleep patterns and dementia, we performed a subgroup analysis in participants younger than 70 years. This analysis is performed only using the Cox regression. The results might be biased because the age limit of 70 implies that the age at which someone could become demented is limited to 70 plus the lag time. Furthermore, we performed a subgroup analysis in participants with a MMSE ≥24, because mild cognitive impairment (MMSE < 24) might also be prodromal dementia [25].

## 3 Results

In Table 2, Table 3, the characteristics of the participants of the first measurement cycle in 1992/3 are described. We found that 17.5% of the participants had difficulty falling asleep, 22.7% had interrupted sleep, 25.2% reported waking up early, 19.1% slept ≤6 h, and 18.3% slept ≥9 h. Participants 70 years and older had more often difficulty falling asleep or waking up early, and a higher percentage slept ≤6 h (23.5 vs 15.8%) or ≥9 h (22.6 vs 15.0) than participants younger than 70 years.

**Table 2:** Descriptive characteristics of participants at baseline (’92-’93).

Sleep Variables	Total %, (n)	Age < 70	Age ≥ 70	Age Group Difference	Follow-up <15 year (incident dementia)	Follow-up ≥ 15 year (incident dementia)	Group Difference
*Hours asleep*							
− 6 h or less	19.1 (356)	15.8 (168)	23.5 (188)	<0.001***	20.4 (30)	32.9 (26)	.038*
− 7 or 8 h	62.6 (1,166)	69.2 (735)	53.9 (431)		53.7 (79)	57.0 (45)	
− 9 h or more	18.3 (340)	15.0 (159)	22.6 (181)	<0.001***	25.9 (38)	10.1 (8)	.005**
*Difficulty falling asleep*							
− almost never / some of the time	82.5 (1,576)	83.9 (901)	80.7 (675)	.064	80.7 (116)	59.0 (49)	.189
− often / most of the time	17.5 (335)	16.1 (173)	19.4 (162)		19.3 (29)	26.8 (22)	
*Interrupted sleep*							
− almost never / some of the time	77.3 (1,291)	78.8 (743)	75.4 (630)	.079	77.3 (116)	59.0 (49)	.003**
− often / most of the time	22.7 (388)	21.3 (206)	24.6 (206)		22.7 (34)	41.0 (34)	
*Waking up early*							
− almost never / some of the time	74.8 (1,426)	77.4 (830)	71.5 (596)	.003**	72.9 (110)	57.3 (47)	.016*
− often / most of the time	25.2 (480)	22.6 (242)	28.5 (238)		27.2 (42)	42.7 (35)	

* significance level *p* < 0.05; ** significance level *p* < .01; *** significance level *p* < .001.

**Table 3:** Descriptive characteristics of participants at baseline (’92 – ’93).

	Hours of sleep ≤6 h	Hours of sleep 7 or 8 h	Hours of sleep ≥9 h	Difficulty falling asleep = no	Difficulty falling asleep = yes	Interrupted sleep = no	Interrupted sleep = yes	Waking up early = no	Waking up early = yes
	*N* = 356	*N* = 1166	*N* = 340	*N* = 1576	*N* = 335	*N* = 1475	*N* = 434	*N* = 1426	*N* = 480
Covariate: Demographics and education									
Age, M (SD)	70.6 (8.7)	67.8 (8.2)	70.7 (7.9)	68.8 (8.3)	69.8 (8.6)	68.8 (8.3)	69.6 (8.7)	68.6 (8.3)	70.2 (8.6)
Female, % (n)	63.4 (225)	50.7 (586)	41.3 (139)	47.7 (747)	73.6 (243)	48.7 (715)	63.9 (274)	49.7 (709)	59.6 (286)
Partner, % (n)	56.9 (202)	75.6 (874)	74.2 (250)	73.5 (1,151)	60.9 (201)	74.3 (1,090)	61.3 (263)	73.9(1,054)	63.3 (303)
Level of education, % (n)									
low	64.8 (230)	55.8 (645)	64.1 (216)	57.4 (900)	69.7 (230)	58.4 (857)	64.1 (275)	56.1 (800)	70.6 (339)
intermediate	24.2 (86)	29.8 (344)	25.8 (87)	28.4 (445)	24.9 (82)	28.0 (410)	26.8 (115)	29.2 (416)	23.1 (111)
high	11.0 (39)	14.5 (167)	10.1 (34)	14.2 (222)	5.5 (18)	13.6 (200)	9.1 (39)	14.7 (210)	6.3 (30)
Other Covariates:									
Physical activity MET's per day, M (SD)	60.8 (38.7)	71.1 (48.7)	59.8 (42.4)	67.6 (46.5)	64.2 (42.2)	68.3 (46.3)	62.5 (43.6)	68.0 (46.5)	63.4 (43.2)
Depressive Symptoms, M (SD)	9.6 (8.9)	6.5 (6.5)	7.3 (7.3)	6.4 (6.3)	11.2 (9.3)	6.2 (6.0)	11.1 (9.4)	6.2 (6.2)	10.4 (9.0)
Diabetes % (n)	5.1 (18)	4.9 (56)	6.5 (22)	5.1 (80)	5.8 (19)	5.1 (75)	5.6 (24)	5.1 (72)	5.8 (28)
Cardiovascular disease, % (n)	19.7 (69)	18.0 (208)	27.8 (93)	19.2 (299)	25.4 (83)	20.0 (292)	21.5 (91)	19.7 (280)	22.3 (106)
Hypertension % (n) ^a^	22.6 (73)	21.7 (230)	21.3 (67)	21.8 (313)	22.7 (69)	22.6 (305)	19.7 (76)	21.1 (276)	24.6 (108)
BMI in ‘92, M (SD)	26.8 (4.0)	26.7 (3.9)	27.1 (4.2)	26.8 (3.9)	27.1 (4.5)	26.8 (4.0)	26.7 (4.2)	26.7 (3.9)	27.2 (4.3)
Smoking, % (n)									
never smoke or > 15 years ago	63.9 (211)	57.7 (645)	54.3 (177)	57.1 (856)	65.7 (209)	56.7 (799)	64.9 (264)	56.9 (778)	63.8 (292)
Stopped < 15 years	17.0 (56)	17.7 (198)	15.0 (49)	18.0 (269)	12.3 (39)	17.4 (245)	15.7 (64)	16.7 (228)	17.9 (82)
Current smoker	19.1 (63)	24.5 (274)	30.7 (100)	24.9 (373)	22.0 (70)	25.9 (365)	19.4 (79)	26.4 (361)	18.3 (84)
Drinking, %, (n)									
Not drinking in ‘92	35.3 115)	28.5 (318)	33.6 (109)	30.4 (453)	36.0 (114)	30.0 (420)	36.4 (148)	29.7 (404)	36.5 (167)
Light alcohol consumption	39.3 (128)	42.8 (477)	43.8 (142)	42.5 (633)	40.1 (127)	43.3 (606)	37.8 (154)	43.5 (591)	37.8 (173)
Heavy alcohol consumption	25.5 (83)	28.7 (320)	22.5 (73)	27.2 (405)	24.0 (76)	26.8 (375)	25.8 (105)	26.8 (364)	25.8 (118)

a. Hypertension is measured in ’96.

Of the 2218 participants, 237 (11%) developed dementia in the period between 1992/3 and 2015/6 (see Fig. 1). Compared to the participants who developed dementia within less than 15 years after the first measurement in 1992/3, a higher percentage of the participants who developed dementia after more than 15 years slept ≤6 h (32.9 vs 20.4%), had interrupted sleep (41.0 vs 22.7%) or woke up early (42.7 vs 27.2%) (Table 2). However, more participants who developed dementia within 15 years slept ≥9 h compared to the participants who developed dementia after more than 15 years (25.9% vs 10.1%).

Of the *N* = 889 participants with missing data, *N* = 573 participants were excluded from the sample because of missing data on sleep disturbances at baseline. These participants were older (71.4 vs 69.0 years, *p* < 0.001), relatively more were female (57% vs 52%) but had a similar risk of incident dementia (15% vs 14%, *p* = .396) compared to the participants who were included.

Table 4 shows the risks of incidence of dementia associated with sleep disturbances in analyses with 7 different lag times (logistic regressions).

**Table 4:** Logistic regression analyses of dementia on sleep variables at baseline and different lag times.

	Hours *^a^* ≤ 6 h ≥ 9 h		Difficulty falling asleep		Interrupted sleep		Waking up early	
Lag time	Model 1	Model 2	Model 1	Model 2	Model 1	Model 2	Model 1	Model 2
Years	OR (95% CI)	OR (95% CI)	OR (95% CI)	OR (95% CI)	OR (95% CI)	OR (95% CI)	OR (95% CI)	OR (95% CI)
3 years	.91 (0.63 – 1.30) 1.51 * (1.07 – 2.13)	.83 (0.56 – 1.23) 1.48* (1.02 – 2.15)	.94 (0.67 –1.31)	.79 (0.52 –1.20)	.78 (0.56 –1.07)	.70 (0.48 –1.02)	.82 (0.60 – 1.12)	.75 (0.52 –1.08)
6 years	1.52 (1.10 – 2.10) 1.05 (0.69 – 1.60)	1.44 * (1.02 – 2.05) 0.90 (0.56 – 1.45)	.92 (0.63 – 1.35)	.94 (0.62 – 1.43)	1.22 (0.90 – 1.66)	1.23 (0.87 – 1.76)	1.44 * (1.07 – 1.94)	1.29 (0.93 – 1.78)
9 years	1.57 * (1.11 – 2.22) 1.11 (0.72 – 1.70)	1.69 ** (1.16 – 2.46) 1.12 (0.70 - 1.79)	1.26 (0.88 – 1.81)	1.45 (0.96 – 2.17)	1.45 * (1.05 – 2.01)	1.63 * (1.11 – 2.38)	1.49 * (1.08 – 2.05)	1.48 * (1.04 – 2.13)
13 years	1.43 (0.96 – 2.13) 1.28 (0.81 – 2.05)	1.45 (0.91 – 2.46) 1.53 (0.92 – 2.55)	1.37 (0.91 – 2.07)	1.29 (0.77 – 2.16)	1.15 (0.78 – 1.70)	1.13 (0.71 – 1.82)	.93 (0.62 – 1.37)	.88 (0.56 – 1.38)
16 years	2.48 *** (1.54 – 3.99) 1.41 (0.76 – 2.61)	2.71 *** (1.57 – 4.68) 1.18 (0.55 – 2.54)	1.81 * (1.09 – 3.01)	1.63 (0.86 – 3.07)	1.92 ** (1.22 – 3.02)	1.66 (0.93 – 2.96)	1.92 ** (1.24 – 2.99)	2.04 ** (1.20 – 3.45)
19 years	2.46 ** (1.26 – 4.80) 0.73 (0.25 – 2.11)	3.44 ** (1.56 – 7.59) 0.74 (0.24 – 2.29)	1.48 (0.69 – 3.19)	1.64 (0.66 – 4.05)	2.38 ** (1.26 – 4.53)	2.97 * (1.26 – 7.03)	3.15 *** (1.77 – 5.62)	3.45 *** (1.71 – 6.93)
23 years	1.83 (0.47 – 7.20) 1.38 (0.30 – 6.78)	2.87 (0.73 – 11.23) 1.48 (0.29 – 7.65)	1.52 (0.45 – 5.17)	2.52 (0.74 –8.56)	4.16 ** (1.52 – 11.40)	7.16 ** (2.09 – 24.51)	7.96 *** (2.60 – 24.41)	10.57 *** (2.86 – 39.01)

*Note.* CI Confidence intervals, OR Odds ratio.

Model 1: corrected for age, sex and education level.

Model 2: additionally corrected for partner, cardiovascular disease, physical activity, depression, smoking, diabetes, drinking, and BMI (all at baseline), and hypertension in ’96.

a. Reference category 7 or 8 h of sleep.

* significance level *p* < 0.05; ** significance level *p* < .01; *** significance level *p* < .001.

For sleeping 9 h or more we found a significant association with an increased risk of dementia only at a lag time of approximately 3 years (the period between two subsequent measurement cycles) (Odds Ratio (OR) = 1.48, 95% confidence interval (CI) = 1.02 – 2.15). With a longer lag time, we found no association between sleeping 9 h and more and incidence of dementia.

For the other sleep disturbance indicators we found that odds ratios increased with longer lag times, except for a lag time of 13 years.

Participants who slept 6 h or less had a 44% higher risk of dementia than participants who slept 7 or 8 h (OR = 1.44 model 2, 95% CI = 1.02 – 2.05) with a lag time of 6 years. The odds ratios increased sharply after a lag time of 16 years, with odds ratios of 2.71 (95% CI = 1.57 – 4.68) and 3.44 (95% CI = 1.56 – 7.59) with a lag time of 19 years for participants who slept 6 h or less.

For interrupted sleep we found an association with incidence of dementia when the lag time was 9 years (OR = 1.63, 95% CI = 1.11 – 2.38). With a lag time of 19 years the OR was 2.97 (95% CI=1.26 – 7.03) and the risk of incidence dementia increased to OR=7.16 (95% CI = 2.09 – 24.51) with a lag time of 23 years.

Waking up early was associated with dementia in the age, education and sex-adjusted model (OR = 1.48, 95% CI = 1.04 – 2.13) with a lag time of 9 years. The risk increased as the lag time was longer: an OR of 2.04 (95% CI=1.20–3.45) if the lag time was 16 years, an OR of 3.45 (95% CI=1.71–6.93) if the lag time was 19 years and an OR of 10.57 (95% CI=2.86 - 39.01) if the lag time was 23 years.

Difficulty falling asleep was not associated with the incidence of dementia in the full model.

### 3.1 Sensitivity analyses

Table 5 shows the risks of incidence of dementia associated with sleep disturbances analyses with 7 different lag times and a subsequent follow-up period of 23.8 years after baseline (Cox analysis).

**Table 5:** Cox analyses of dementia on sleep variables at baseline (’92 – ’92) and different lag times.

	Hours *^a^* ≤ 6 h ≥ 9 h		Difficulty falling asleep		Interrupted sleep		Waking up early	
Range lag times ^b^	Model 1	Model 2	Model 1	Model 2	Model 1	Model 2	Model 1	Model 2
Years	HR (95% CI)	HR (95% CI)	HR (95% CI)	HR (95% CI)	HR (95% CI)	HR (95% CI)	HR (95% CI)	HR (95% CI)
2.2 – 23.8	1.28 (0.93 – 1.78) 1.40 (1.00 – 1.98)	1.32 (0.92 – 1.89) 1.36 (0.092 – 2.00)	1.17 (0.85 –1.61)	1.14 (0.78 –1.64)	1.31 (0.99 –1.74)	1.29 (0.92 –1.79)	1.41 * (1.07 – 1.86)	1.51 * (1.10 –2.07)
5.5 – 23.8	1.48 * (1.05 – 2.09) 1.32 (0.89– 1.96)	1.47 * (1.01 – 2.15) 1.36 (0.89 – 2.07)	1.28 (0.91 – 1.80)	1.27 (0.86 – 1.87)	1.43 * (1.05 – 1.94)	1.42 * 1.00 – 2.02	1.63 ** (1.21 – 2.20)	1.61 ** (1.15 – 2.24)
8.4 – 23.8	1.39 (0.95 – 2.04) 1.35 (0.88 – 2.06)	1.45 (0.96 – 2.19) 1.40 (0.89 - 2.20)	1.32 (0.91 – 1.91)	1.34 (0.89 – 2.02)	1.40 (1.00 – 1.96)	1.47 * (1.01 – 2.14)	1.45 * (1.04 – 2.03)	1.49 * (1.03 – 2.14)
12.5 – 23.8	1.34 (0.87 – 2.07) 1.34 (0.83 – 2.16)	1.52 (0.97 – 2.40) 1.49 (0.89 – 2.20)	1.35 (0.89 – 2.05)	1.34 (0.89 – 2.13)	1.50 * (1.03 – 2.17)	1.51 (1.00 – 2.28)	1.41 (0.97 – 2.05)	1.62 * (1.09 – 2.42)
15.6 – 23.8	2.31 ** (1.41 – 3.77) 0.92 (0.43 – 1.95)	2.80 *** (1.65 – 4.73) 1.05 (0.48 – 2.28)	1.69 * (1.02 – 2.80)	1.69 (0.97 – 2.94)	2.29 *** (1.47 – 3.56)	2.12 ** (1.29 – 3.49)	2.49 *** (1.59 – 3.92)	2.73 *** (1.69 – 4.40)
18.8 – 23.8	2.49 ** (1.27 – 4.90) 0.68 (0.20 – 2.27)	3.30 ** (1.60 – 6.81) 0.77 (0.22 – 2.65)	1.23 (0.58 – 2.61)	1.41 (0.63 – 3.18)	3.00 *** (1.66 – 5.44)	3.37 ** (1.70 – 6.71)	3.98 *** (2.13 – 7.42)	4.32 *** (2.22 – 8.40)
22.5 – 23.8	1.79 (0.49 – 6.54) 1.38 (0.29 – 6.44)	2.76 (0.68 – 11.13) 1.43 (0.28 – 7.37)	1.49 (0.47 – 4.68)	2.39 (0.63 –9.18)	3.76 ** (1.45 – 9.77)	6.37 ** (2.00 – 20.27)	7.23 *** (2.52 – 20.75)	9.35 *** (2.83 – 30.84)

*Note.* CI Confidence intervals, HR Hazard ratio.

Model 1: corrected for sex and education level.

Model 2: additionally corrected for partner, cardiovascular disease, physical activity, depression, smoking, diabetes, drinking, and BMI (all at baseline), and hypertension in ’96.

a. Reference category 7 or 8 h of sleep.

b. The follow-up periods range from the baseline cycle till the ’15 -’16 interview cycle.

* significance level *p* < 0.05; ** significance level *p* < .01; *** significance level *p* < .001.

We find that in the Cox regressions the effects fluctuate less and are statistically significant with a shorter lag time than in the main analyses. For example, taking all follow-up cycles into account, participants who woke up early also had a higher risk of incident dementia than those who did not (HR=1.51, 95% CI = 1.10 – 2.07). And participants with interrupted sleep had a higher risk of incidence of dementia with a lag time of ≥5.5 years (HR=1.42, 95% CI = 1.00–2.02). The relative risks of dementia generally increased with longer lag times. Most notable were the risks when the lag time was more than 15 years. For example, the relative risk of dementia for participants who slept ≤6 h was 2.80 (95% CI = 1.65 – 4.73, model 2) when the lag time was ≥15.6 years and 3.30 (95% CI = 1.60 – 6.81) when the lag time was ≥18.8 years.

When people 70 years or older were excluded from the sample, 103 (11%) of the 949 remaining baseline participants developed dementia in the period from 1992/3 to 2015/6 (Table A (Annex)). Compared to the Cox analysis with all participants, we find an association with incident dementia only with lag times less than 12.8 years for waking up early. However, results for other indicators of sleep quality were similar.

Restricting the analyses to the participants with a MMSE ≥ 24 at baseline (*N* = 1612), we found similar results as in the main Cox analysis (results in Annex, Table B). However, no association was found of a sleep duration of ≥9 h or more anymore.

We further checked the results of the analysis with the Cox analysis if we maximized the lag time to 9 years (the maximum of 3 interview cycles). With this lag time, we did not find an association between sleep disturbances and the incidence of dementia, as opposed to longer lag times (Annex, Table C).

## 4 Discussion

In this study, we investigated the association of sleep disturbances at baseline with incident dementia at follow-up, with a maximum follow-up time of 23.8 years. We explored the effect of varying the lag time to dementia incidence to take reverse causation into account.

We found that the lag time had a major effect on the outcomes. Sleeping 9 h or more was associated with incident dementia when the lag time was 3 years, whereas we found no association of sleeping ≥9 h with incident dementia when the lag time was more than 5.5 years. Furthermore, the relative risks of dementia generally increased with longer lag times. For lag times of 6 years and longer, we found positive associations of interrupted sleep and waking up early with risk of dementia. Most notable are the risks when the lag time was 16 years or more, with strong associations between interrupted sleep, waking up early and incident dementia.

If sleeping ≥9 h is causally related to the incidence of dementia, we would expect equal or stronger associations of sleeping ≥9 h with dementia with longer lag times. Further insight in the association of a sleep duration of ≥9 h with dementia was provided by the Cox analyses. We did not find an association of a sleep duration of ≥9 h with incident dementia, first, with a younger (<70 years) and, second, with a cognitively healthier sample (MMSE ≥24). The presence of this association in the full sample might be due to changes in the sleep patterns in the preclinical and prodromal phase. The possible reverse causation is in line with Westwood et al. [26] who find that prolonged sleep duration was associated with an increased risk of incident dementia in a study with much shorter follow-up of 10 years. Therefore, it is more likely that having a sleep duration of ≥9 h is rather a symptom of than a contributor of dementia.

Our results contribute to the few studies in the literature with a long follow-up period. Some previous findings were in line with ours. No association of a sleep duration of ≥9 h and dementia was found as well by Lutsey et al. [9], who had a follow-up period up to 16 years and Sabia et al. [10]. Sabia et al. [10] followed cohorts aged 50, 60 and 70 years old with a mean of 24.6, 14.8 and 7.5 years follow-up. However, other studies seem to contradict our findings [11,12]. Sindi et al. [12] combined data from three studies, with late life baseline analyses based on data with a follow-up of 3–10 years and midlife baseline analyses with a follow-up of 21 and 32 years. They found that a long sleep duration (>9 h) was associated with an increased dementia risk. This result was based however on a shorter, 5–9 years, follow-up. A study by Virta et al. [11] had a median follow-up time of 22.5 years and showed that long (a sleep duration of > 8 h) sleepers had lower cognitive scores than participants sleeping 7–8 h a day. In contrast to Virta et al. [11], we were able to provide more insight in timing of incidence of dementia, because we investigated the effect of a change in the lag time.

Lutsey et al. [9] found that sleeping < 7 h was associated with all cause dementia. Sabia et al. [10] found that less than 6 h of sleep was associated with dementia. However, the effects were small: HRs ranged from 1.22 to 1.37. Compared to Sabia et al. [10], our effect estimates were much larger. Our results support findings by Sindi et al. [12] on the association of dementia and insomnia and waking up early. The results on midlife insomnia were based on long-term follow-up, whereas the results on waking up early were based on 5–10 years of follow-up.

A limitation of our study is that we use self-reported data on sleep disturbances. Various studies found that self-reported data on sleep duration overestimate the number of hours of sleep: by 0.8 h with 6 h of sleep measured by actigraphy and 0.4 h with 7 h of sleep [27] or 20–30 min compared to polysomnography and actigraphy [28]. Regardless of these seemingly modest deviations of the self-reported sleep duration, future research should give more insight in the validation of other self-reported sleep disturbances such as waking up early and interrupted sleep.

A strength of this study is the long-follow up period in combination with the multiple measurement cycles, that allowed us to investigate the association of sleep disturbances and dementia for different lag times. This method might give more insight in contradicting or inconclusive results in other cohort studies with varying lag times. Furthermore, with longer lag times the risk of a bias in the sample with relatively cognitively more healthy participants is smaller than with shorter lag times.

Our results partly confirm earlier results of the association of sleep disturbances with incident dementia. The novel contribution of our study is that it gives more insight into the impact of varying lag times and the age of the study sample. Future research should provide more insight into the question whether changes in sleep pattern after midlife are related to ageing per se or to the prodromal phase of dementia. Our results stress the importance of a high sleep quality during midlife.

## Funding

The Longitudinal Aging Study Amsterdam is supported by a grant from the Netherlands Ministry of Health Welfare and Sports, Directorate of Long-Term Care (https://www.government.nl/ministries/ministry-ofhealth-welfare-and-sport) [grant number N/A].

## Contributions

Peter Alders: Drafting/revision of the manuscript for content, including medical writing for content; Study concept or design; Analysis or interpretation of data; Almar Kok: Drafting/revision of the manuscript for content, including medical writing for content; Study concept or design; Analysis or interpretation of data; Elisabeth M. Zutphen: Drafting/revision of the manuscript for content, including medical writing for content; Analysis or interpretation of data; Jurgen Claassen: Drafting/revision of the manuscript for content, including medical writing for content; Analysis or interpretation of data; Dorly J.H. Deeg: Drafting/revision of the manuscript for content, including medical writing for content; Study concept or design; Analysis or interpretation of data.

## Declaration of competing interest

All authors declare that they have no conflicts of interest.

## Appendix Supplementary materials

Image, application 1
