# Supplementary material for: The effect of sleep disturbances on the incidence of dementia for varying lag times
Source: J Prev Alzheimers Dis. 2025 Jan 1;12(2):100024. doi: 10.1016/j.tjpad.2024.100024 (PMC12184010; doi:10.1016/j.tjpad.2024.100024)
Supplement: Supplementary file 1 [file mmc1.docx]

***Annex***

**Table A:** Cox analyses of dementia on sleep variables at baseline and different lag times with age < 70 years at baseline (’92 – ’93).

|  | **Hours ^a^**  <=6 hours  >=9 hours |  | **Difficulty falling**  **asleep** |  | **Interrupted sleep** |  | **Waking up early** |  |
| --- | --- | --- | --- | --- | --- | --- | --- | --- |
| **Range lag times ^b^** | **Model 1** | **Model 2** | **Model 1** | **Model 2** | **Model 1** | **Model 2** | **Model 1** | **Model 2** |
| Years | HR (95% CI) | HR (95% CI) | HR (95% CI) | HR (95% CI) | HR (95% CI) | HR (95% CI) | HR (95% CI) | HR (95% CI) |
| 2.2 – 23.8 | 1.42  (.87 – 2.31)  1.29  (.74 – 2.26) | 1.53  (.90 – 2.60)  1.17  (.62 – 2.22) | 1.13  (.69 – 1.87) | 1.06  (.59 – 1.90) | 1.41  (.93 – 2.15) | 1.33  (.81 – 2.19) | 1.80 **  (1.20 – 2.70) | 1.94 **  (1.24 – 3.04) |
| 5.5 – 23.8 | 1.39  (.84 – 2.29)  1.26  (.71 – 2.24) | 1.53  (.90 – 2.61)  1.18  (.62 – 2.23) | 1.17  (.71 – 1.93) | 1.05  (.59 – 1.89) | 1.40  (.92 – 2.16) | 1.33  (.81 – 2.20) | 1.82 **  (1.21 – 2.76) | 1.95 **  (1.24 – 3.06) |
| 8.4 – 23.8 | 1.35  (.81 – 2.24)  1.20  (.67 – 2.17) | 1.50  (.87 – 2.58)  1.23  (.65 – 2.33) | 1.16  (.69 – 1.93) | 1.00  (.55 – 1.82) | 1.48  (.96 – 2.27) | 1.40  (.85 – 2.31) | 1.84 **  (1.21 – 2.79) | 1.92 **  (1.22 – 3.04) |
| 12.8 – 23.8 | 1.33  (.78 – 2.26)  .85  (.42 – 1.74) | 1.49  (.84 – 2.63)  .92  (.43 – 1.94) | 1.06  (.61 – 1.86) | .98  (.52-1.84) | 1.61 *  (1.03 – 2.53) | 1.55  (.92 – 2.61) | 1.85 **  (1.18 – 2.89) | 2.04 **  (1.26 – 3.30) |
| 15.6 – 23.8 | 1.95 *  (1.12 – 3.42)  .43  (.13 – 1.42) | 2.24 **  (1.22 – 4.10)  .46  (.14 – 1.56) | 1.09  (.57 – 2.07) | 1.02  (.50 – 2.10) | 1.89 *  (1.13 – 3.16) | 1.77  (.98 – 3.20) | 2.69 ***  (1.61 – 4.49) | 2.86 ***  (1.66 - 4.92) |
| 18.8 – 23.8 | 2.14 *  (1.01 – 4.49)  .27  (.04 – 2.01) | 2.52 *  (1.12 – 5.66)  .27  (.03 – 2.07) | .93  (.38 – 2.29) | 1.03  (.39 – 2.78) | 2.75 **  (1.42 – 5.31) | 3.03 **  (1.40 – 6.55) | 4.33 ***  (2.19 – 8.55) | 4.74 ***  (2.27 – 9.90) |
| 22.5 – 23.8 | 1.67  (.46 – 6.10)  .76  (.09 – 6.05) | 2.70  (.64 – 11.28)  .80  (.09 – 6.94) | 1.13  (.31 – 4.10) | 1.75  (.39 – 7.85) | 3.71 *  (1.34 – 10.26) | 8.22 **  (2.29 – 29.52) | 7.48 ***  (2.49 – 22.48) | 11.28 ***  (3.13 – 40.68) |

*Note.* CI Confidence intervals, HR Hazard ratio

Model 1: corrected for age, sex and education level.

Model 2: additionally corrected for partner, cardiovascular disease, physical activity, depression, smoking, diabetes, drinking, and BMI (all at baseline), and hypertension in ’96.

a. Reference category 7 or 8 hours of sleep.

b. The follow-up periods range from the baseline cycle till the ’15 -’16 interview cycle.

* significance level p <.05; ** significance level p < .01; *** significance level p < .001

**Table B:** Cox analyses of dementia on sleep variables at baseline and different lag times with MMSE ≥ 24 at baseline (’92 – ’93).

|  | **Hours ^a^**  <=6 hours  >=9 hours |  | **Difficulty falling**  **asleep** |  | **Interrupted sleep** |  | **Waking up early** |  |
| --- | --- | --- | --- | --- | --- | --- | --- | --- |
| **Range lag times ^b^** | **Model 1** | **Model 2** | **Model 1** | **Model 2** | **Model 1** | **Model 2** | **Model 1** | **Model 2** |
| Years | HR (95% CI) | HR (95% CI) | HR (95% CI) | HR (95% CI) | HR (95% CI) | HR (95% CI) | HR (95% CI) | HR (95% CI) |
| 2.2 – 23.8 | 1.29  (.91 – 1.82)  1.20  (.82 – 1.77) | 1.31  (.90 – 1.91)  1.24  (.82 – 1.88) | 1.25  (.89 – 1.76) | 1.17  (.80 – 1.73) | 1.40 *  (1.03 – 1.90) | 1.31  (.93 – 1.86) | 1.48 *  (1.10 – 2.00) | 1.54 *  (1.11 – 2.15) |
| 5.5 – 23.8 | 1.49 *  (1.05 – 2.16)  1.20  (.79 – 1.84) | 1.43  (.99 – 2.14)  1.25  (.80 – 1.94) | 1.42  (.99 – 2.02) | 1.36  (.92 – 2.02) | 1.54 **  (1.12 – 2.11) | 1.44 *  (1.01 – 2.07) | 1.63 **  (1.19 – 2.24) | 1.58 *  (1.12 – 2.23) |
| 8.4 – 23.8 | 1.42  (.96 – 2.10)  1.22  (.78 – 1.93) | 1.47  (.97 – 2.23)  1.25  (.78 – 2.02) | 1.40  (.95 – 2.04) | 1.39  (.91 – 2.11) | 1.50 *  (1.06 – 2.12) | 1.50 *  (1.02 – 2.20) | 1.46 *  (1.03 – 2.06) | 1.51 *  (1.04 – 2.20) |
| 12.5 – 23.8 | 1.39  (.90 – 2.16)  1.11  (.65 – 1.90) | 1.54  (.97 – 2.45)  1.25  (.72 – 2.17) | 1.42  (.93 – 2.17) | 1.36  (.85 – 2.19) | 1.55 *  (1.06 – 2.27) | 1.50  (.99 – 2.29) | 1.47 *  (1.00 – 2.17) | 1.65*  (1.10 – 2.49) |
| 15.6 – 23.8 | 2.28 **  (1.39 – 3.76)  .95  (.44 – 2.02) | 2.84 ***  (1.67 – 4.85)  1.09  (.50 – 2.36) | 1.74 *  (1.04 – 2.89) | 1.77 *  (1.01 – 3.10) | 2.26 ***  (1.45 – 3.54) | 2.14 **  (1.29 – 3.54) | 2.47 ***  (1.57 – 3.89) | 2.72 ***  (1.68 – 4.40) |
| 18.8 – 23.8 | 2.53 **  (1.29 – 4.96)  .70  (.21 – 2.33) | 3.32 **  (1.61 – 6.87)  .79  (.23 – 2.70) | 1.24  (.58 – 2.63) | 1.40  (.62 – 3.15) | 3.03 ***  (1.67 – 5.50) | 3.39 **  (1.70 – 6.75) | 4.01 ***  (2.15 – 7.48) | 4.31 ***  (2.22 – 8.39) |
| 22.5 – 23.8 | 1.77  (.48 – 6.49)  1.37  (.29 – 6.40) | 2.73  (.68 – 11.02)  1.43  (.28 – 7.31) | 1.48  (.47 – 4.66) | 2.38  (.62 – 9.12) | 3.74 **  (1.44 – 9.71) | 6.32 **  (1.99 – 20.12) | 7.19 ***  (2.50 – 20.62) | 9.27 ***  (2.81 – 30.60) |

*Note.* CI Confidence intervals, HR Hazard ratio

Model 1: corrected for age, sex and education level.

Model 2: additionally corrected for partner, cardiovascular disease, physical activity, depression, smoking, diabetes, drinking, and BMI (all at baseline) and hypertension in ’96.

a. Reference category 7 or 8 hours of sleep.

b. The follow-up periods range from the baseline cycle till the ’15 -’16 interview cycle.

* significance level p <.05; ** significance level p < .01; *** significance level p < .001

**Table C:** Cox analyses of dementia on sleep variables at baseline and different lag times at baseline (’92 – ’93).

|  | **Hours ^a^**  <=6 hours  >=9 hours |  | **Difficulty falling**  **asleep** |  | **Interrupted sleep** |  | **Waking up early** |  |
| --- | --- | --- | --- | --- | --- | --- | --- | --- |
| **Range lag times ^b^** | **Model 1** | **Model 2** | **Model 1** | **Model 2** | **Model 1** | **Model 2** | **Model 1** | **Model 2** |
| Years | HR (95% CI) | HR (95% CI) | HR (95% CI) | HR (95% CI) | HR (95% CI) | HR (95% CI) | HR (95% CI) | HR (95% CI) |
| 2.2 – 9.0 | 1.22  (.75 – 1.99)  1.38  (.84 – 2.26) | 1.13  (.63 – 2.04)  1.24  (.68 – 2.26) | 1.02  (.62 – 1.68) | .87  (.46 – 1.64) | 1.08  (.69 – 1.69) | .95  (.53 – 1.69) | 1.33  (.88 – 2.02) | 1.28  (.76 – 2.15) |
| 12.5 – 23.8 | 1.34  (.87 – 2.07)  1.34  (.83 – 2.16) | 1.52  (.97 – 2.40)  1.49  (.89 – 2.20) | 1.35  (.89 – 2.05) | 1.34  (.89 – 2.13) | 1.50 *  (1.03 – 2.17) | 1.51  (1.00 – 2.28) | 1.41  (.97 – 2.05) | 1.62 *  (1.09 – 2.42) |

*Note.* CI Confidence intervals, HR Hazard ratio

Model 1: corrected for age, sex and education level.

Model 2: additionally corrected for partner, cardiovascular disease, physical activity, depression, smoking, diabetes, drinking, and BMI (all at baseline) and hypertension in ’96.

a. Reference category 7 or 8 hours of sleep.

b. The follow-up periods range from the baseline cycle till the ’15 -’16 interview cycle.

* significance level p <.05; ** significance level p < .01; *** significance level p < .001
